# Effects of Nickel Impregnation on the Catalytic Removal of Nitric Oxide by Polyimide-Based Activated Carbon Fibers

**DOI:** 10.3390/nano13162297

**Published:** 2023-08-10

**Authors:** Hun-Seung Jeong, Byung-Joo Kim

**Affiliations:** 1 Material Application Research Institute, Jeonju University, Jeonju 55069, Republic of Korea; hunseung.jeong@jj.ac.kr; 2 Department of Energy Science, Sungkyunkwan University, Suwon 16419, Republic of Korea; 3 Department of Advanced Materials and Chemical Engineering, Jeonju University, Jeonju 55069, Republic of Korea

**Keywords:** polyimide-based activated carbon fiber, catalysts, metal, nitric oxide

## Abstract

Activated carbon fibers (ACFs) are beneficial for adsorbing harmful gases because of the well-developed micropores on their surface. Usually, the physical adsorption of harmful gases by ACFs is limited by their textural properties. In this study, the effect of nickel particle catalyst impregnation on the physicochemical removal of nitric oxide (NO) by polyimide (PI)-based ACFs (PI-ACFs) was investigated. Ni(NO_3_)_2_ was used as the precursor of nickel particle catalysts and impregnated on ACFs as a function of concentrations. The Ni(NO_3_)_2_/ACFs were then thermally reduced in an argon atmosphere containing 4% hydrogen (400 °C, 1 h). The gases generated during heat treatment were verified using Fourier transform infrared spectroscopy, and the impregnation amount of metallic nickel was also calculated based on the gas amount generated. The specific surface areas of the ACF and Ni-ACFs were determined to be 1010–1180 m^2^/g, while the nickel impregnation amount was 0.85–5.28 mg/g. The NO removal capacity of the Ni-ACF was found to be enhanced with the addition of Ni catalysts. In addition, metallic nickel particles on the ACFs maintained their chemical molecular structures before and after the NO removal tests.a

## 1. Introduction

Industrialization has led to air pollution, a severe environmental problem primarily caused by particulate emissions such as coal dust, ozone, carbon dioxide, volatile hydrocarbons, and nitrogen oxides. Among these, NOx are considered major atmospheric pollutants, contributing to various environmental problems such as acid rain, photochemical smog, and ozone layer destruction [1,2]. Although types of NOx include NO, NO_2_, NO_3_, N_2_O_3_, N_2_O_4_, and N_2_O_5_, the term NOx generally refers to NO and NO_2_. At room temperature, the equilibrium between NO and NO_2_ is favored toward NO_2_, and the NO produced by combustion is oxidized in the atmosphere to form NO_2_ [3]. Therefore, removing NO before its emission into the atmosphere is crucial to reducing NO and NO_2_. Previous studies have been conducted on NOx purification technologies [4,5,6], and dry and wet methods have been used to remove NOx. In the case of the dry method, selective catalytic reduction (SCR) by NH_3_, selective noncatalytic reduction (SNCR) and adsorption by adsorbents have been used [7,8]. Meanwhile, in the case of the wet method, a scrubber column is used, in which NOx is absorbed by the absorbent [9,10]. Among the aforementioned methods, the most widely used NOx reduction technology is SCR by NH_3_. However, this process is limited due to the high reaction temperature (>300 °C) and leakage of NH_3_. Moreover, additional costs are incurred to reheat the desulfurization gas or replace deactivated catalysts. Therefore, an inexpensive method that removes NOx stably at low temperatures (<150 °C) is required for NOx removal.

Methods for removing NOx at low temperatures include the use of activated carbons (ACs) and their fibers (ACFs) [11,12]. These porous carbons are beneficial owing to their low cost [13,14], resistance to acids and bases [15], wide specific surface area [16,17,18], porous structure [19,20], and high catalytic activity [21]. However, NOx removal using only these adsorbents is limited, and much research has been conducted to introduce various metals and additives to porous carbon carriers. The commonly used metals include Cu [22], Mn [23], Ni [24,25], Fe [26], Co [27], and Ru [28], which are impregnated on the surface of porous carbon, thereby enhancing its adsorption capacity by acting as catalysts. Khristova et al. [29] prepared nickel-impregnated AC (Ni-AC) by impregnating AC in a solution of Ni(NO_3_)_2_, followed by heat treatment at 200–300 °C; the content of nickel was 2.9–8.3%. The produced Ni-AC was reported to have an approximately fourfold enhanced NO conversion capacity in an air atmosphere, ranging from 80 to 350 °C. Yamashita et al. [30] prepared nickel-impregnated carbon samples by impregnating coal particles in a solution of nickel (II) acetate, followed by heat treatment at 650 °C. The reported nickel content was approximately 4.0%, and the samples exhibited approximately twice the NO conversion capacity in an air atmosphere at 300 °C. Santiago Veiga et al. [31] prepared nickel-impregnated carbon samples by impregnating coal particles in a solution of nickel (II) acetate and maintaining the suspension with stirring for 5 h. Then, the solvent was removed by rotary evaporation, and the obtained solid was dried in an oven at 110 °C for 24 h, followed by heat treatment at 700 °C under argon flow (20 mL/min) for 6 h. The reported nickel content was approximately 10%. Numerous studies [29,30,31] have used AC as a porous carrier, primarily impregnated with more than 3% nickel. It has been reported that the specific surface area of nickel-impregnated carbon materials decreases by up to 30%. However, studies utilizing ACFs as porous carriers have not been exhaustively conducted. Compared with ACs, ACFs have well-developed micropores on their surfaces. Furthermore, the porous characteristics of the ACFs enable fast adsorption and desorption, resulting in excellent adsorption even at low concentrations [32,33]. Thus, ACFs can serve as better porous carriers than ACs. However, the micropores distributed on their surfaces can be filled or blocked by metals and additives [34,35].

ACFs are typically manufactured using various precursors [36,37,38,39], including polyacrylonitrile, cellulose, and pitch. The characteristics of the precursor influence several fundamental properties of ACFs, including specific surface area, pore structure, surface characteristics, adsorption properties, and yields [35,36,37,38,39]. Recently, ACFs utilizing polyimide (PI) are gaining attention for their economic advantages owing to a process characteristic that can skip a stabilization step [40]. However, no studies have been conducted on metal impregnation and harmful gas removal using PI-based ACFs (PI-ACFs).

This study investigated the metallic nickel impregnation of the PI-based ACFs within a range that did not significantly reduce the specific surface area and their effects on the NO removal. The amount of nickel on the ACFs was controlled to be <1 wt.% to minimize the impact on the pore structure with varying nickel content. Changes in the surface functional groups and NO-removing ability corresponding to the nickel content were evaluated. Additionally, changes in the chemical molecular structure of metallic nickel were observed by comparing the results before and after NO adsorption. 

## 2. Experimental

### 2.1. Materials

The PI fibers used in this study were supplied by Dissol Co., Ltd. (Jeonju, Republic of Korea). The PI fibers (7.5 g) were placed in an alumina crucible and carbonized using a custom quartz tube furnace (SiC heater, 1000 mm × 90 mm) under an atmosphere of ultrahigh-purity nitrogen (N_2_, 99.999%, 200 cc/min). Carbonization was maintained at 800 °C (heating rate 10 °C/min) for 60 min, following which the fibers were allowed to cool to room temperature (25 °C). The carbonized fibers (CFs) were weighed (3.7 g), which confirmed a carbonization yield of 49.3%.

For activation, the CFs (3.5 g) were placed in a boat-type alumina crucible and loaded into a tubular furnace (Inconel, 1200 mm × 80 mm). An ultrahigh-purity nitrogen atmosphere (99.999%, 200 cc/min) was maintained until the activation temperature was reached (900 °C, heating rate 10 °C/min), following which it was replaced with steam (0.5 mL/min) upon reaching the activation temperature; activation was performed for 30 min. Subsequently, the atmosphere was maintained under ultrahigh-purity nitrogen during the natural cooling phase. The resultant ACF was named PI-ACF.

For nickel impregnation, the PI-ACFs (1.0 g) were placed in a square dish, and impregnation solutions of 0.10, 0.50, 1.00, 5.0, and 9.0 mol of Ni(NO_3_)_2_·6H_2_O were used. Each concentration of the solution was sprayed onto the PI-ACF using a spray bottle, with 0.2 mL of the Ni(NO_3_)_2_·6H_2_O solution being sprayed per 1.0 g of the PI-ACF. The impregnated PI-ACF was dried at 110 °C for 24 h and then heat-treated at 400 °C for 1 h under an argon atmosphere (200 cc/min) with 4% hydrogen to obtain the reduced metallic catalyst. After heat treatment, all samples were stored in a vacuum oven (0.1 Pa). The gases generated during the heat treatment were examined using Fourier transform infrared spectroscopy (FT-IR, I4001-E, MIDAC Corp., Westfield, NJ, USA). The content of nickel-impregnated on the ACF was determined through Inductively Coupled Plasma Optical Emission Spectroscopy (ICP-OES, Spectro ARCOS, SPECTRO Analytical Instruments LTD., Kleve, Germany). The prepared PI-ACFs were named 0.1, 0.5, 1.0, 5.0, and 9.0-Ni-ACF.

### 2.2. Characterization

The surface metal of the ACFs was identified using X-ray diffractometry (XRD; SmartLab SE, Rigaku Co., Tokyo, Japan) within the range of 10–60° at a scan speed of 2°/min using Cu-Kα as the light source (λ = 1.54 Å). The presence and distribution of specific elements in the ACFs were confirmed using X-ray photoelectron spectroscopy (XPS; Nexsa XPS system, Thermo Fisher Scientific Inc., Waltham, MA, USA). The X-ray anode was operated in a vacuum chamber at 2.0 × 10^−7^ torr over the binding energy range of 0–1300 eV. O_1S_ and Ni_2P_ data were obtained to observe the chemical binding states of oxygen and nickel present on the surface of the ACs.

The pore characteristics of the ACFs were measured using an isothermal adsorption analyzer (BELSORP-Max II, Microtrac BEL, Osaka, Japan) and analyzed using the obtained N_2_/77K adsorption-desorption isotherm. The specific surface area of the ACF was calculated using the Brunauer-Emmett-Teller (BET) equation [41], and the volume of the micropores and pore size distribution were calculated using the t-plot [42] and non-local density functional theory (NLDFT) methods [43], respectively. The mesopore volume was obtained by subtracting the micropore volume from the total pore volume.

### 2.3. NO Removal (Adsorption)

Before the adsorption analysis of NO, all samples were dried in a vacuum oven (0.1 Pa) at 110 °C for 24 h and then filled to a certain volume of 0.4 g in a reaction tube (quartz column). The NO removal experiment was conducted by introducing 30 ppm NO (200 cc/min, N_2_ balance) after purging with N_2_ for 1 h. The adsorption capacity is expressed relative to the initial concentration (C/C_0_ = 1.0 (30 ppm)). The experiment was terminated when the outlet concentration reached 30 ppm. The concentration of NO was detected by FT-IR, which is capable of gas analysis, and the chemical composition changes in the Ni-ACF before and after the adsorption were confirmed using XRD.

## 3. Results and Discussion

### 3.1. Preparation of Nickel-Impregnated ACF Samples

**Figure 1:**
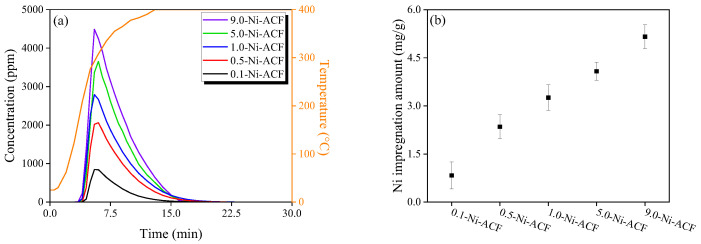
(**a**) NO generation amount from Ni-impregnated activated carbon fiber as a function of Ni(NO_3_)_2_ concentration with reduction temperature and (**b**) calculated Ni amount based on the result of (**a**).

The Ni(NO_3_)_2_-impregnated ACF was reduced by heat treatment, and the gases generated during the heat treatment process were confirmed by FT-IR. Figure 1a illustrates the gases generated by heat treatment, and the detected curve represents NO, which is the decomposition gas of Ni(NO_3_)_2_. All ACFs detected NO near 200 °C, and the detected amount was observed to have increased with the impregnated amount of Ni(NO_3_)_2_. Figure 1b shows the amount of impregnated Ni calculated from the detected amount of NO. The amount of impregnated Ni (*Ni_imp_* mg/g) was calculated using Equations (1) and (2).

Ni(NO_3_)_2_ + 4H_2_ → Ni + 2NO + 4H_2_O (1)

(2) $${Ni}_{imp}=\frac{{Ni}_{weight}}{{2NO}_{weight}}\times {NO}_{det}$$

where *Ni_weight_* is the atomic weight of nickel (g/mol), *NO_weight_* is the molecular weight of NO (g/mol), and *NO_det_* is the amount of NO (mg/g) generated by thermal decomposition. The results confirmed that the impregnated nickel amounts of 0.1-, 0.5-, 1.0-, 5.0-, and 9.0-Ni-ACF were 0.83, 2.35, 3.25, 4.08, and 5.16 mg/g, respectively.

Table 1 presents the nickel content of Ni-impregnated ACF, which was determined through ICP-OES analysis, providing the actual amount of nickel adsorbed. The nickel content for 0.1-, 0.5-, 1.0-, 5.0-, and 9.0-Ni-ACF was found to be 0.56, 2.10, 2.89, 3.88, and 5.41 mg/g, respectively. Notably, the calculated nickel content from FT-IR and the confirmed nickel content from ICP-OES exhibit remarkably similar trends, validating the accuracy of the measurements. However, it is essential to note that there is a slight difference in nickel content between FT-IR and ICP-OES, which can be attributed to the method of nickel (Ni(NO_3_)_2_) impregnation via spray. This variation is considered the influencing factor responsible for the minor discrepancy observed between the two measurements. Overall, both FT-IR and ICP-OES analyses served as valuable tools in confirming the nickel content of Ni-impregnated ACF in this study.

### 3.2. XRD and XPS Analyses

**Figure 2:**
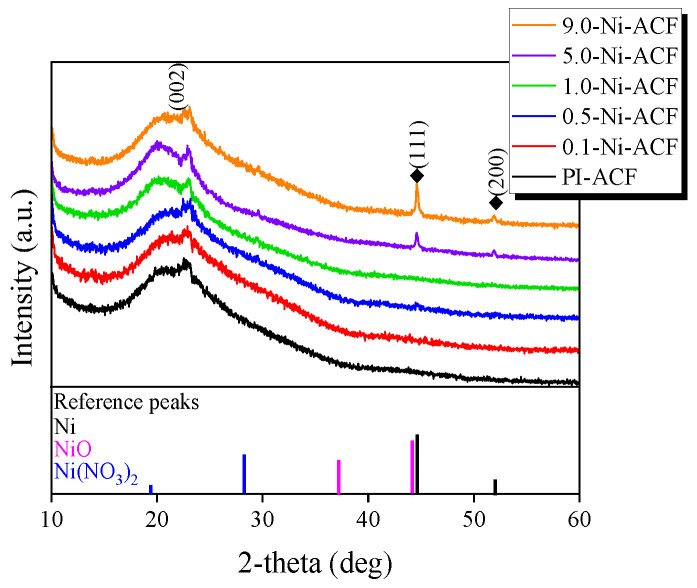
XRD patterns of nickel-impregnated activated carbon fibers as a function of Ni(NO_3_)_2_ impregnation concentration.

Figure 2 exhibits the XRD patterns of the Ni-impregnated ACFs. XRD analysis is a helpful method for analyzing the crystal structure of ACF and confirming the chemical structure of impregnated Ni [44]. In the untreated ACF, a wide non-crystalline carbon structure was observed at 20–25°, resulting from the diffraction from the (002) plane owing to the irregularly stacked structure of graphite. A significantly broad peak was found around 43°, composed of not clearly separated (100) and (101) planes due to the incomplete graphite molecular layers. After nickel impregnation and subsequent thermal reduction, a leftward bias of the (002) plane was observed, which was similar to the increase in the interlayer spacing due to the oxidation of the fine crystal structure by oxygen molecules generated during Ni(NO_3_)_2_ decomposition. Moreover, as the amount of impregnation increased, the peak intensities of 44.6° and 51.9° increased. The peaks at 44.6° and 51.9° correspond to the (111) and (200) planes of the FCC crystal structure of pure nickel [45]. Thus, the impregnated material was primarily composed of metallic nickel. Peaks corresponding to the reference NiO and Ni(NO_3_)_2_ were not observed in any samples.

**Figure 3:**
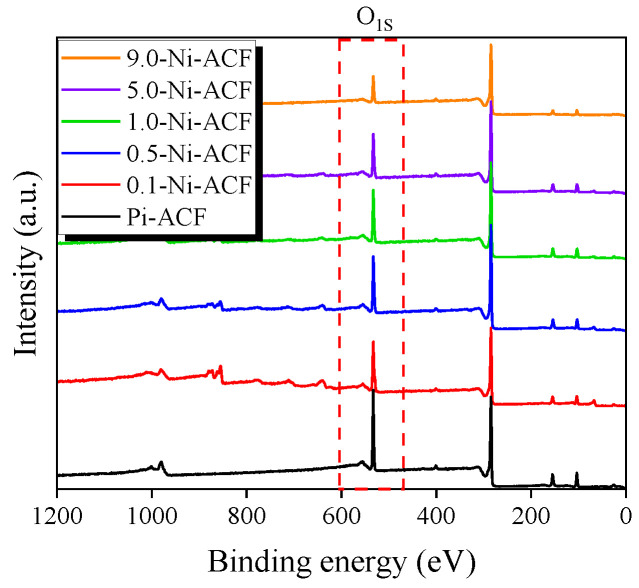
XPS survey spectra of nickel-impregnated activated carbon fibers as a function of Ni(NO_3_)_2_ impregnation concentration at room temperature.

XPS is a non-destructive (or weak) surface technique that utilizes the electron binding energy of atoms present on the sample surface to determine elemental composition and chemical state. Figure 3 illustrates the change in oxygen content in Ni-ACF following nickel impregnation and subsequent thermal reduction treatment. Although all Ni-ACF samples were treated at the same temperature and time, the oxygen content tended to decrease as the nickel content increased. It is believed that the deposited nickel acted as a catalyst for the reduction reaction, enhancing the activation energy and reaction rate, and thus, more vigorous reduction occurred as the nickel content increased.

**Figure 4:**
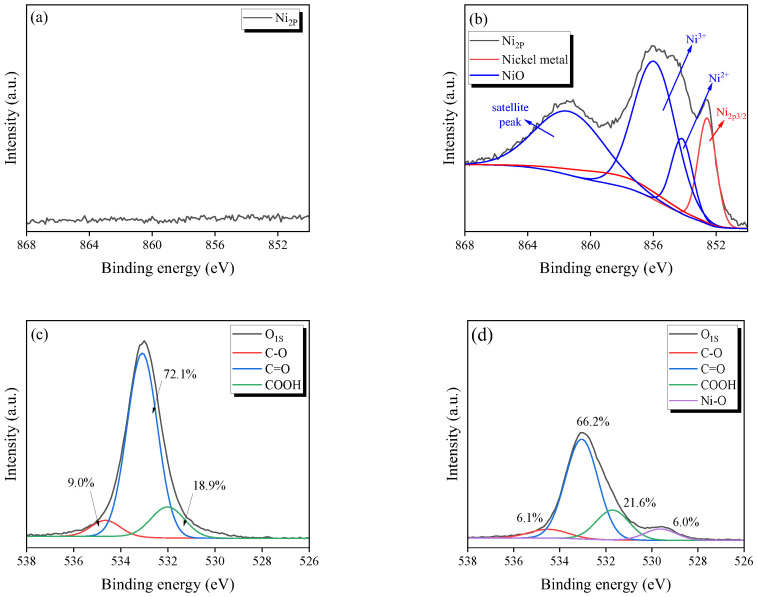
High-resolution Ni_2P_ and O_1S_ deconvolution peaks of nickel-impregnated activated carbon fibers; (**a**) Ni_2P_ of PI-ACF, (**b**) Ni_2P_ of 1.0-Ni-ACF, (**c**) O_1S_ of PI-ACF, and (**d**) O_1S_ of 1.0-Ni-ACF.

Figure 4 presents the O_1S_ and Ni_2P_ spectra of the Ni-ACFs obtained using XPS. Figure 4a,b show the Ni_2P_ peaks of the untreated PI-ACF and 1.0-Ni-ACF, respectively, where Ni_2P_ and O_1S_ are compared on the same y-axis scale. While no Ni peak was discernible in the untreated PI-ACF, Ni metal and NiOx peaks in the 850–868 eV range were detected in the 1.0-Ni-ACF due to the impregnation and reduction processes [46]. Figure 4c,d show the O_1S_ peaks of the untreated PI-ACF and 1.0-Ni-ACF, respectively. For the PI-ACF, peaks for C-O, C=O, and COOH were observed at 9.0%, 72.1%, and 18.9%, respectively, while those for the 1.0-Ni-ACF were observed at 6.1%, 66.2%, and 21.6%, with an additional Ni-O peak at 6.0%. All the oxygen functional groups decreased after reduction, with the decrease in C-O being the most obvious. XPS and XRD verified the impregnation state of nickel on the ACF, and most of the nickel was confirmed to be pure metallic nickel by XRD. Furthermore, the XPS results confirmed that some of the nickel on the surface was formed as NiO.

### 3.3. N_2_/77K Adsorption-Desorption Isotherm Curve

**Figure 5:**
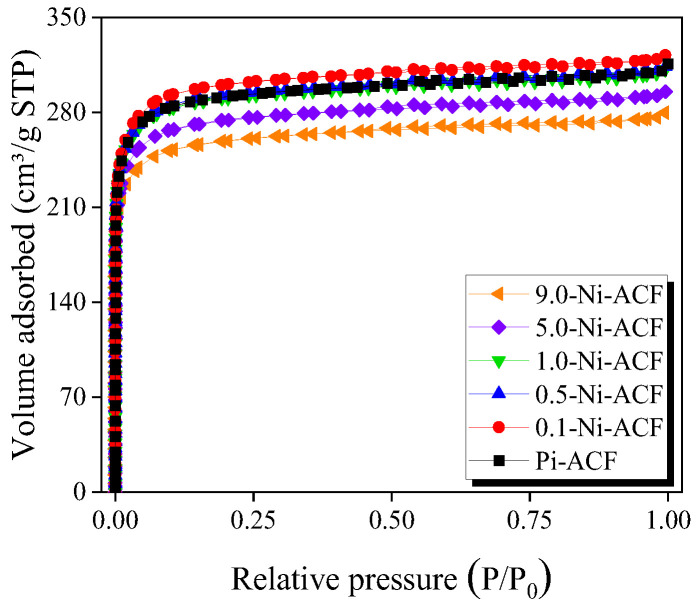
N_2_/77K adsorption-desorption isotherm curves of nickel-impregnated activated carbon fibers as a function of Ni(NO_3_)_2_ impregnation concentration.

Figure 5 exhibits the N_2_/77K adsorption-desorption isotherms of the ACF. All ACFs were classified as Type I according to the IUPAC classification [47], and their N_2_ adsorption was predominantly observed at a relative pressure (P/P_0_) of <0.1. This indicates monolayer adsorption owing to the strong interaction between the pore walls of the ACF and N_2_, suggesting that the ACFs are primarily microporous. The decrease in adsorption at a relative pressure (P/P_0_) of <0.1 with increased impregnated nickel was confirmed. However, despite impregnation, increased N_2_ adsorption was observed at a relative pressure (P/P_0_) of <0.1 in the 0.1-Ni-ACF, and a subsequent decrease was confirmed with an increase in the amount of impregnated nickel. This may be because the amount of pore opening due to (1) the decomposition of oxygen functional groups and (2) the oxidation of ultrafine crystallites by additionally generated oxygen molecules, as confirmed by XRD, was more significant than the reduction in adsorption caused by pore blocking due to nickel impregnation. However, an increase in additional impregnation led to the dominant occurrence of typical pore blocking.

The shape of the hysteresis loop correlates with specific pore characteristics [47]. The N_2_/77K adsorption-desorption isotherms of all the ACF samples exhibited type H4 hysteresis according to the IUPAC classification. Even with increased impregnation, the area of the hysteresis loop did not change significantly. This indicates that the untreated ACF possessed slit-shaped pores and that the impregnation and thermal reduction treatments did not significantly alter the pore shape.

**Figure 6:**
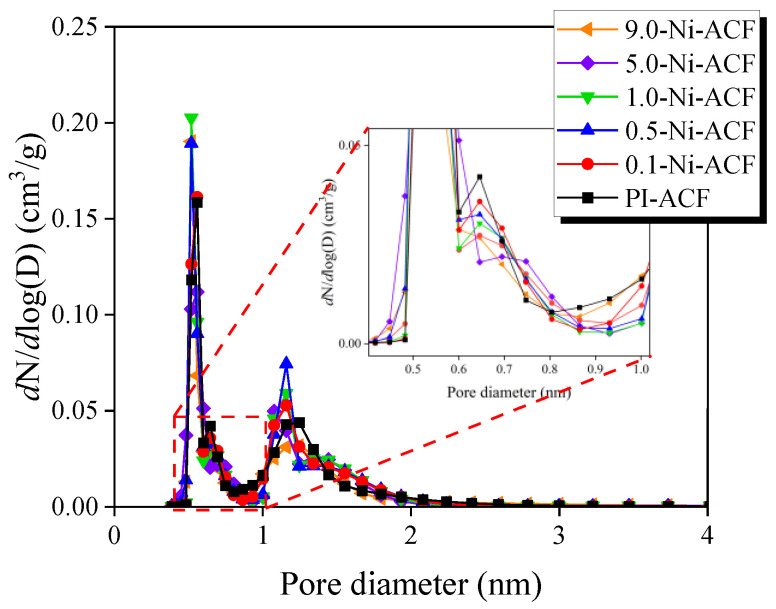
Pore size distribution of the nickel-impregnated activated carbon fibers as a function of Ni(NO_3_)_2_ impregnation concentration.

Figure 6 illustrates the pore size distribution (PSD) curves for Ni-ACF, which were obtained using the NLDFT equation. In Figure 6, the PSD curves for Ni-ACFs display a gradual decrease in pore volume for sizes below 1.0 nm as the amount of nickel impregnation increases. However, in the range of 1.0–2.0 nm, the pore volume shows an increase from Pi-ACF to 0.5-Ni-ACF, followed by a subsequent decrease up to 9.00-Ni-ACF. Notably, there are no significant changes in pore volume observed for mesopores with sizes of 2.0 nm and above. These changes in pore volume are attributed to the influence of (1) the decomposition of oxygen functional groups and (2) the oxidation of ultrafine crystallites by additionally generated oxygen molecules. This has resulted in an increase in pore volume at certain pore sizes and a decrease in pore volume in the range of 1.0–2.0 nm as the nickel impregnation amount increases.

### 3.4. Textural Properties

Table 2 presents the pore characteristics of the ACF as a function of nickel impregnation. The specific surface area and total pore volume of the ACF were 1010–1180 m^2^/g and 0.43–0.50 cm^3^/g, respectively. The micropore volume of the ACF was 0.41–0.47 cm^3^/g, while its fractional micropore volume was 94.0–94.7%. Compared to the untreated ACF, the nickel impregnated ACF exhibited a gradual decrease in specific surface area. However, in the case of 0.1 and 0.5-Ni-ACF, the oxygen functional groups were observed to have been removed, and additional mesopores were opened because of the thermal reduction treatment, thus increasing the specific surface area. As the amount of impregnation increased, the specific surface area decreased. The micropore volume exhibits a trend similar to that of the specific surface area. The mesopore volume was 0.03 cm^3^/g for all samples except 9.0-Ni-ACF.

### 3.5. NO Adsorption Behavior

**Figure 7:**
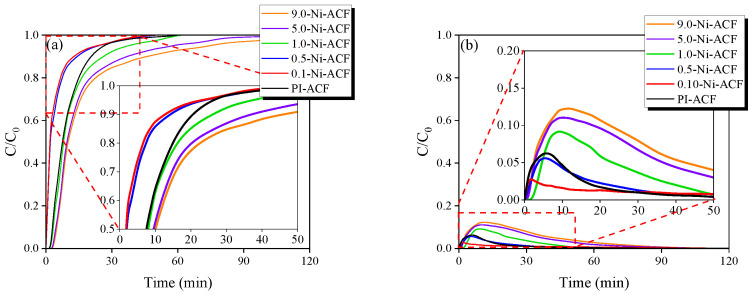
(**a**) NO adsorption curves and (**b**) CO_2_ generation curves of nickel-impregnated activated carbon fibers as a function of Ni(NO_3_)_2_ impregnation concentration at room temperature.

Figure 7a illustrates the NO adsorption behavior of the ACF, where C/C_0_ = 1.0 (30 ppm) is the saturation point. The adsorption capacity up to saturation was in the order of 0.1-Ni-ACF < 0.5-Ni-ACF < PI-ACF < 1.0-Ni-ACF < 5.0-Ni-ACF < 9.0-Ni-ACF. The specific surface area of the ACF was in the order of 9.0-Ni-ACF < 5.0-Ni-ACF < 1.0-Ni-ACF < PI-ACF < 0.5-Ni-ACF < 0.1-Ni-ACF; NO adsorption showed a completely opposite behavior to the specific surface area. Although the nickel content followed the order 0.1-Ni-ACF < 0.5-Ni-ACF < 1.0-Ni-ACF < 5.0-Ni-ACF < 9.0-Ni-ACF, it did not correlate directly with the NO adsorption capacity. Therefore, the NO adsorption behavior was influenced by a combination of the specific surface area (physical adsorption) and nickel content (chemical adsorption), along with other potential variables.

Figure 7b shows the CO_2_ detection curve according to NO adsorption. CO_2_ was detected in all ACF samples in the order of 0.1-Ni-ACF < 0.5-Ni-ACF < PI-ACF < 1.0-Ni-ACF < 5.0-Ni-ACF < 9.0-Ni-ACF. The behavior of CO_2_ detection was parallel to the trend observed for the NO adsorption capacity. This similarity in the behavior of NO adsorption and CO_2_ detection suggests the emission of CO_2_ during the adsorption of NO on carbonaceous materials. The following equation represents the NO adsorption mechanism on carbonaceous surfaces [48,49].

(3) $$2NO+{Ni}_{ACF}+C_{ACF}\to CO+N_{2}+{NiO}_{ACF}$$

(4) $$CO+{NiO}_{ACF}\;\to {CO}_{2}+{Ni}_{ACF}$$

**Figure 8:**
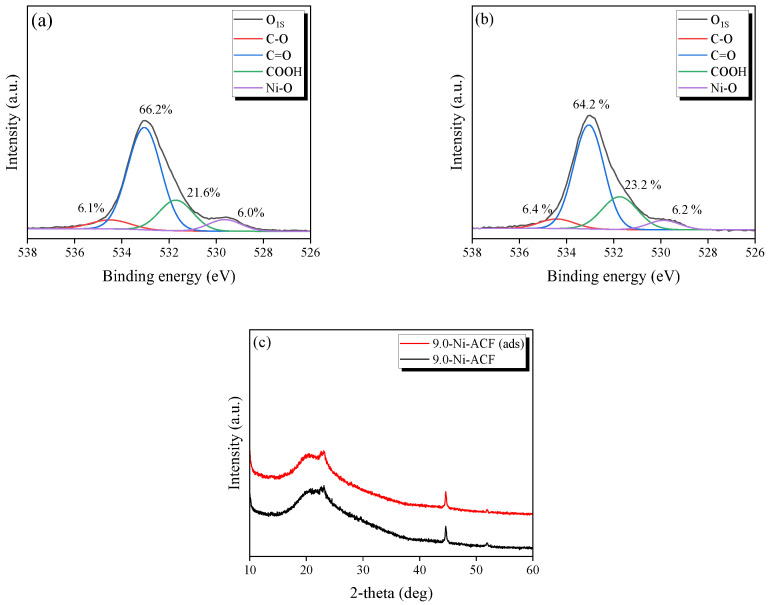
High-resolution O_1S_ deconvolution peaks of nickel-impregnated activated carbon fibers before and after NO adsorption: (**a**) 9.0-Ni-ACF before NO adsorption, (**b**) 9.0-Ni-ACF. (**c**) XRD pattern comparison of 9.0-Ni-ACF before and after NO adsorption.

Figure 8 presents the XPS O_1S_ spectra before and after NO adsorption for the 9.0-Ni-ACF sample and the XRD results before and after adsorption for the untreated PI-ACF and 9.0-Ni-ACF samples. As shown in Figure 8b, no NO peak associated with physical adsorption was observed. The percentages of C-O, C=O, COOH, and Ni-O before adsorption were 6.1%, 66.2%, 21.6%, and 6.0%, and after adsorption were 6.4%, 64.2%, 23.2%, and 6.2%. Interestingly, only the quantity of C=O decreased, whereas those of the others increased. This can be attributed to the higher initial proportion of C=O in the PI-ACF, where a higher probability of conversion to COOH rather than C=O occurs during the oxidation of the carbon surface. Although the increase in Ni-O after the adsorption treatment was 0.2%, it was assumed to be within the range of experimental error. This suggests that impregnated nickel primarily plays a catalytic role. Furthermore, Figure 8c illustrates the XRD patterns before and after adsorption, which show no evident differences, confirming the presence of pure Ni peaks at 44.6° and 51.9°. These results indicate that the nickel-impregnated on ACF undergoes only catalytic interactions without significant chemical transformations during NO adsorption.

**Figure 9:**
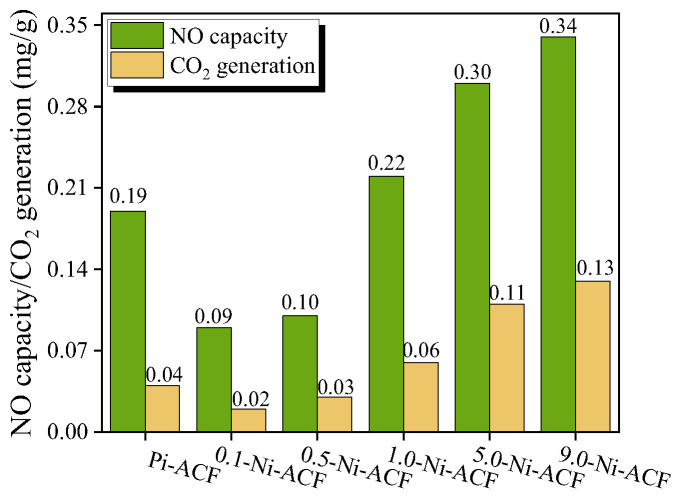
NO removal and CO_2_ generation capacity of nickel-impregnated activated carbon fibers as a function of Ni(NO_3_)_2_ impregnation concentration at room temperature.

We illustrate the NO adsorption capacity and CO_2_ emission in Figure 9, which shows similar trends. Compared to PI-ACF, the NO adsorption capacity and CO_2_ emissions decreased for 0.1-Ni-ACF and 0.5-Ni-ACF and then increased with higher nickel loadings. Notably, despite nickel impregnation, the NO adsorption capacity and CO_2_ emission decreased for these samples, which have higher specific surface areas than the untreated PI-ACF. This suggests that factors other than nickel impregnation contributed to the reduction in the adsorption capacity. This decrease in NO adsorption capacity and CO_2_ emission, despite the impregnation of nickel, suggests that factors other than nickel loading led to a decrease in the adsorption capacity. These specific samples exhibited a higher surface area than the untreated PI-ACF, indicating that other factors contributed to the observed reduction in adsorption capacity.

Figure 3 shows the oxygen content based on the XPS analysis for all ACF samples, indicating a decreasing trend for all heat-reduced samples compared with the untreated sample. In the NO adsorption behavior of the adsorbent, physical adsorption is initially performed, and then chemical/catalytic adsorption is performed. Therefore, the decrease in surface oxygen functional groups can lead to a decrease in the amount of physical adsorption in the initial stage, which can directly cause a decrease in the amount of chemical/catalytic adsorption in the later stage. In particular, the observed decrease in NO adsorption for the 0.1-Ni-ACF and 0.5-Ni-ACF samples, despite the presence of nickel as a catalyst and their high surface areas, can be attributed to the reduction of oxygen functional groups during the thermal reduction process [50,51]. This reduction in oxygen functional groups can weaken the dipole-dipole interaction between the ACF surface and NO molecules, thus decreasing the adsorption of polar NO molecules. For samples with higher nickel content, starting from 1.0-Ni-ACF, the catalytic reaction of nickel became more active, resulting in increased adsorption capacity compared to the untreated PI-ACF. However, as the adsorption capacity continued to increase, the blockage of the pore structure intensified, leading to a further decrease in the initial material adsorption and ultimately decreasing the overall NO adsorption removal characteristics.

## 4. Conclusions

In this study, we investigated the NO adsorption removal behavior of nickel-impregnated PI-based ACFs and found that it was influenced by the amount of impregnated nickel and surface oxygen functional groups. The impregnated metallic nickel played a catalytic role without undergoing significant chemical structure changes in composition, whereas the adsorption removal of NO increased with increasing nickel impregnation within a range in which the specific surface area did not decrease significantly. Furthermore, due to the reduction treatment, the surface oxygen content decreased, and samples with lower nickel loadings exhibited lower NO adsorption removal than the untreated ACF. However, compared to the untreated sample, a distinct increase in NO adsorption was observed with a nickel loading of 0.3 wt.% or higher. Nevertheless, excessive nickel impregnation is expected to further block the pore structure and negatively affect NO adsorption removal. The unchanged pore diameter due to this impregnation is in the nanoscale range, indicating that the impregnated metal particles are expected to be in the nanoscale or even smaller size, as the pore structure remains unaltered after impregnation. In addition, a minor metal impregnation method that does not significantly affect the pore structure could offer various contributions to future nanomaterial research. This approach can be applied not only to metals other than nickel but also to diverse research fields, such as energy storage technologies (e.g., activated carbon in supercapacitors), beyond the field of adsorption. Therefore, we will conduct further research to investigate the optimal impregnation loading, focusing on nanoscale impregnation studies for our future investigations.

## Figures and Tables

**Table 1:** Amount of Ni-Impregnated by Reduction of Ni(NO_3_)_2_ using FT-IR and Amount of Ni-Impregnated by ICP-OES.

Sample Name	FT-IR (mg/g)	ICP-OES (mg/g)
PI-ACF	-	-
0.1-Ni-ACF	0.83	0.56
0.5-Ni-ACF	2.35	2.10
1.0-Ni-ACF	3.26	2.89
5.0-Ni-ACF	4.08	3.88
9.0-Ni-ACF	5.16	5.41

**Table 2:** Textural Properties of Ni-impregnated Polyimide-based Activated Carbon Fibers as a Function of Ni(NO_3_)_2_ Concentration.

Sample Name	S_BET_^a^ (m^2^/g)	V_Total_^b^ (cm^3^/g)	V_Micro_^c^ (cm^3^/g)	V_Meso_^d^ (cm^3^/g)	R_Micro_^e^ (%)	D_avg_^f^ (nm)
PI-ACF	1150	0.48	0.45	0.03	93.8	1.69
0.1-Ni-ACF	1180	0.50	0.47	0.03	94.0	1.68
0.5-Ni-ACF	1160	0.49	0.46	0.03	93.9	1.71
1.0-Ni-ACF	1130	0.48	0.45	0.03	93.8	1.71
5.0-Ni-ACF	1070	0.46	0.43	0.03	93.5	1.73
9.0-Ni-ACF	1010	0.43	0.41	0.02	95.3	1.74

^a^ S_BET_: Specific surface area: BET method $\frac{p}{v(p_{0}-p)}=\frac{1}{v_{m}}+\frac{c-1}{v_{m}c}\cdot \frac{p}{p_{0}}$. ^b^ V_Total_: Total pore volume; The amount adsorbed P/P_0_ = 0.99. ^c^ V_Micro_: Micropore volume: t-plot methods. ^d^ V_Meso_: ^b^ V_Total_− ^c^ V_Micro_. ^e^ R_Micro_: Micropore volume ratio; $\frac{V_{Micro}}{V_{Total}}\times 100$ ^f^ D_avg_: Average pore diameter.

## Data Availability

Not applicable.
